# Biomimetic Composite Coatings for Activation of Titanium Implant Surfaces: Methodological Approach and In Vivo Enhanced Osseointegration

**DOI:** 10.3390/mi12111352

**Published:** 2021-10-31

**Authors:** Daniel Oltean-Dan, Gabriela-Bombonica Dogaru, Elena-Mihaela Jianu, Sorin Riga, Maria Tomoaia-Cotisel, Aurora Mocanu, Lucian Barbu-Tudoran, Gheorghe Tomoaia

**Affiliations:** 1 Department of Orthopedics and Traumatology, Iuliu Hatieganu University of Medicine and Pharmacy, 47 General Traian Mosoiu Street, 400132 Cluj-Napoca, Romania; olteandandaniel@yahoo.com; 2 Department of Medical Rehabilitation, Iuliu Hatieganu University of Medicine and Pharmacy, 46-50 Viilor Street, 400347 Cluj-Napoca, Romania; dogarugabrielaumf@gmail.com; 3 Department of Histology, Iuliu Hatieganu University of Medicine and Pharmacy, 6 Louis Pasteur Street, 400349 Cluj-Napoca, Romania; me05doc@yahoo.com; 4 Research Center of Physical Chemistry, Department of Chemical Engineering, Faculty of Chemistry and Chemical Engineering, Babes-Bolyai University, 11 Arany Janos Street, 400028 Cluj-Napoca, Romania; d_s_riga@yahoo.com (S.R.); mocanu.aurora@gmail.com (A.M.); 5 Academy of Romanian Scientists, 54 Splaiul Independentei, 050085 Bucharest, Romania; 6 Electron Microscopy Laboratory Prof. C. Craciun, Faculty of Biology and Geology, Babes-Bolyai University, 5-7 Clinicilor Street, 400006 Cluj-Napoca, Romania; lucian.barbu@ubbcluj.ro

**Keywords:** osseointegration, modified titanium implants, biomimetic composite coating, layer-by-layer method, bone remodeling, bone regeneration, bone–implant contact

## Abstract

Innovative nanomaterials are required for the coatings of titanium (Ti) implants to ensure the activation of Ti surfaces for improved osseointegration, enhanced bone fracture healing and bone regeneration. This paper presents a systematic investigation of biomimetic composite (BC) coatings on Ti implant surfaces in a rat model of a diaphyseal femoral fracture. Methodological approaches of surface modification of the Ti implants via the usual joining methods (e.g., grit blasting and acid etching) and advanced physicochemical coating via a self-assembled dip-coating method were used. The biomimetic procedure used multi-substituted hydroxyapatite (ms-HAP) HAP-1.5 wt% Mg-0.2 wt% Zn-0.2 wt% Si nanoparticles (NPs), which were functionalized using collagen type 1 molecules (COL), resulting in ms-HAP/COL (core/shell) NPs that were embedded into a polylactic acid (PLA) matrix and finally covered with COL layers, obtaining the ms-HAP/COL@PLA/COL composite. To assess the osseointegration issue, first, the thickness, surface morphology and roughness of the BC coating on the Ti implants were determined using AFM and SEM. The BC-coated Ti implants and uncoated Ti implants were then used in Wistar albino rats with a diaphyseal femoral fracture, both in the absence and the presence of high-frequency pulsed electromagnetic shortwave (HF-PESW) stimulation. This study was performed using a bone marker serum concentration and histological and computer tomography (micro-CT) analysis at 2 and 8 weeks after surgical implantation. The implant osseointegration was evaluated through the bone–implant contact (BIC). The bone–implant interface was investigated using FE-SEM images and EDX spectra of the retrieved surgical implants at 8 weeks in the four animal groups. The obtained results showed significantly higher bone–implants contact and bone volume per tissue volume, as well as a greater amount of newly formed bone, in the BC-coated Ti implants than in the uncoated Ti implants. Direct bone–implant contact was also confirmed via histological examination. The results of this study confirmed that these biomimetic composite coatings on Ti implants were essential for a significant enhancement of osseointegration of BC-coated Ti implants and bone regeneration. This research provides a novel strategy for the treatment of bone fractures with possible orthopedic applications.

## 1. Introduction

Osseointegration is the direct contact between living bone and the surface of a load-bearing synthetic implant, without the interposition of non-bone tissue [1]. To achieve osseointegration, various metals, ceramics and polymers are used. Major metal types that are used to achieve osseointegration are titanium and its alloys, cobalt, tantalum, stainless steel and zirconium. Titanium (Ti) was recognized worldwide as a biocompatible material that has high mechanical properties, resistance to corrosion under a biological environment and the ability to develop a dynamic oxide layer on the surface [2,3]. However, Ti metal does not possess an osteoconductive property on its own [4].

The host bone response to a titanium implant is influenced by the roughness, porosity, and topography of the Ti surface. The surface morphology directly influences the osteoblast and osteoclast attachment and metabolism [5]. There are also host-dependent factors that influence osseointegration in terms of the host bone quality, such as bone mineral density (BMD), bone mineral content (BMC), vascular integrity of the bone, mechanical loading conditions and bone type (cortical or cancellous). Nevertheless, the immune system still considers titanium implants as foreign bodies and fibrosis around the implants can occur. In this way, the osteoblasts are restricted to creating bone ingrowth at the surface of the implant and osseointegration is constrained. This condition leads to aseptic loosening and failure of the implant [6]. The limitation of fibrous tissue production can be achieved by a bioactive coating of the Ti implant surface with a hydroxyapatite (HAP) bioceramic, which can lead to benefits in terms of implant fixation [7,8,9,10]; the adhesion, proliferation and differentiation of osteoblasts [11]; and bone matrix formation. Basically, HAP is considered to be an osteoconductive material. It is also known that the surface roughness of the implant surface is an important factor, which might modulate the activity of osteoblasts [12,13] and can affect in vivo bone apposition and the mechanical strength of the implant–bone interface [7]. Accordingly, improved osseointegration was associated with an increased surface roughness [14,15,16,17,18,19]. Although the hydroxyapatite-coated implants had a significantly higher surface roughness compared to grit-blasted or chemical-etched implants, it has not been conclusively revealed whether the surface roughness or the hydroxyapatite coating was the dominant factor that affected the in vivo osseointegration.

Furthermore, the bioactive performance of bioceramic materials was improved by substituting various elements that are essential for bone regeneration, such as Mg, Zn or Si [20,21,22,23,24,25,26], into the HAP lattice, resulting in multi-substituted HAP (ms-HAP) bioceramics. The interest in this substitution originated from the well-established role of these elements in bone regeneration [27,28]. The ms-HAP materials are similar to the main mineral phase in bones in terms of their chemical composition and structure and their role was recently shown to enhance the medical performance of Ti implants in clinical orthopedic applications [4,26]. However, the application of HAP bioceramics is limited due to their brittle nature, which can cause fragile failure during implantation [29]. This type of drawback can be overcome by using a composite of HAP or ms-HAP bioceramics and a polymeric matrix, such as polylactic acid (PLA), which is a famous synthetic biodegradable polymer [30] for medical applications, particularly in bone fixation and surgical sutures [30,31,32,33]. However, PLA has disadvantages because it does not have any bone conductivity and its acidic degradation products could be harmful to the human body [34]. It is documented that the incorporation of HAP nanoparticles in the PLA matrix leads to HAP@PLA composites, which combine the osteoconductivity and bone-bonding properties of HAP bioceramics with the rather easy processing properties of PLA. Furthermore, HAP or ms-HAP materials can be used to delay the early PLA biodegradation and neutralize its acidic degradation, enhancing the mechanical performance of the obtained composite material. It was reported that HAP@PLA composites show great potential for clinical applications due to their good bioactivity, absorbability and degradation properties [35,36,37].

According to our previous work, increased interaction between bioceramic material and PLA can be achieved by using ms-HAP, synthesized in the presence of collagen (COL) dispersion as ms-HAP/COL core/shell nanoparticles (NPs) [26]. These NPs were embedded into a PLA matrix, obtaining a porous composite, namely, ms-HAP/COL@PLA, which was deposited on the surface of a Ti implant to produce a controlled surface topography by using a layer-by-layer (LBL) self-assembly deep-coating method. Finally, the porous composite coating layer was covered with an adsorbed COL layer, obtaining the biomimetic composite (BC) ms-HAP/COL@PLA/COL, which was deposited on Ti implants. Previously, the role of this biomimetic composite deposited on Ti implants was evaluated in vivo and its performance was demonstrated regarding the enhanced bone consolidation with or without high-frequency pulsed electromagnetic short-waves (HF-PESW) biophysical stimulation using rat femoral fractures.

To the best of our knowledge, this study is the first in vivo investigation that focused on the osseointegration of this biomimetic coating on a Ti implant surface in a rat model. For the in vivo evaluation of the osseointegration, two types of Ti implants were used, namely, standard (uncoated) Ti (STi) and BC-coated STi (BCSTi) implants in rat femoral fractures, with or without HF-PESW biophysical stimulation. It should be kept in mind that the surface roughness of the implants and the chemical composition of the coating on implants are important factors that affect osseointegration in vivo. Therefore, this study was designed to investigate how these factors contributed to the osseointegration of the orthopedic-relevant Ti surface. The study hypothesis was that the addition of a biomimetic composite coating, based on multi-substituted hydroxyapatite, collagen and PLA, on Ti implants would enhance the osseointegration beyond that provided by a change in surface roughness alone. For this purpose, the surface roughness of the modified Ti implants was chosen to be in the same nanoscale, between about 200 nm for (uncoated) standard Ti (STi) implants, up to about 600 nm for biomimetic composite (BC)-coated STi (BCSTi) implants. Considering that the reported thickness of the coating on a Ti surface ranged from 448 nm [4] to over 1 µm, for example, about 50 µm [7], a selected thickness of the coating of about 1.5 µm was used in this investigation. Our present in vivo study showed for the first time that the biomimetic composite coating jointly containing the multi-substituted HAP (i.e., HAP-1.5 wt% Mg-0.2 wt% Zn-0.2 wt% Si), PLA and collagen enhanced the osseointegration beyond what was provided by the change in surface roughness alone in Wistar albino rats. In addition, the role of HF-PESW biophysical stimulation in the early 2 weeks from the implantation was also evaluated.

## 2. Materials and Methods

### 2.1. Materials and Methods

The three modified Ti implants were prepared as follows: the surfaces of the Ti implants were first improved via grit-blasting with P500 and labeled Ti implants; subsequently, these Ti implants were chemically activated using acid etching with 50 wt% phosphoric acid solution for 10 min, leading to the standard (control) Ti implants, called STi implants, which had a controlled surface roughness. The Ti surface was cleaned using an ultrasonication process and sterilized, as shown previously [26]. Then, the STi implants were further coated using the biomimetic composite (BC), which was a multifunctional ms-HAP/COL@PLA/COL coating that was deposited using a layer-by-layer self-assembly method on the STi implants, resulting in the BCSTi (coated) implants.

The ms-HAP was a three-substituted HAP, containing Mg, Zn and Si, particularly HAP-1.5 wt% Mg-0.2 wt% Zn-0.2 wt% Si. The ms-HAP was functionalized with COL as HAP-1.5 wt% Mg-0.2 wt% Zn-0.2 wt% Si/6%COL (core/shell nanoparticles) and incorporated into the PLA matrix, resulting in HAP-1.5 wt% Mg-0.2 wt% Zn-0.2 wt% Si/COL@PLA/COL, which was a biomimetic composite enriched in Mg.

Briefly, to prepare this biomimetic composite coating on the STi implants, a two-phased approach was used. First, a porous ms-HAP/COL@PLA biomimetic composite (PBC) was deposited on the STi surface as an intermediate layer from mixed dispersions of ms-HAP/COL and PLA of known composition, resulting in the PBC-STi implants. Finally, on this porous biomimetic composite PBC, an outermost collagen layer that was formed from self-assembled COL fibrils was adsorbed, resulting in ms-HAP/COL@PLA/COL, which was called the BC coating on STi implants. For the in vivo osseointegration evaluation of this BC coating, two types of Ti implants were used, namely, STi and BCSTi implants in rat femoral fractures, with and without HF-PESW.

### 2.2. Characterization Methods of the Ti Implants and Composite Coatings

A Hitachi SU-8230 field emission scanning electron microscope (FE-SEM, also called SEM) was used to explore the nanostructure of both the biomimetic composites and the bone–implant interface. The FE-SEM was equipped with an Oxford energy-dispersive X-ray spectrometer for elemental analysis (energy-dispersive X-ray (EDX) spectra). SEM samples were prepared by depositing the biomimetic composite ms-HAP/COL@PLA/COL on a Ti (plate) surface, which had the same surface characteristics as the STi (rods) implants. The FE-SEM and EDX spectra were also used for the surface morphology and elemental analysis of the newly formed bone on the retrieved implants at 8 weeks after the implantation surgery.

Atomic force microscopy (AFM) images were obtained using AFM JEOL 4210 equipment that was operated in tapping mode using standard cantilevers with silicon nitride tips (resonant frequency in the range of 200–300 kHz and spring constant of 17.5 N/m) [38,39,40,41,42]. The surface roughness was determined using AFM on the Ti (plate) surface as follows: Ti—Ti surface was grit-blasted; STi—Ti surface was grit-blasted and acid-etched; PBC-STi—STi surface was coated with the porous biomimetic composite ms-HAP/COL@PLA; BCSTi—STi surface was coated with the biomimetic composite (BC), having adsorbed an outermost COL layer, namely, ms-HAP/COL@PLA/COL. The biomimetic coatings were deposited from their dispersions on the Ti (plate) surface, with the same surface characteristics as STi (rods). The surface roughness parameters of the Ti plates, which were uncoated, covered with the porous biomimetic composite ms-HAP/COL@PLA or covered with the biomimetic composite ms-HAP/COL@PLA/COL, were expressed as the arithmetic mean Ra and root mean square Rq (RMS) [41].

### 2.3. Surgical Protocol and Procedures

The present study was performed according to the recommendations of the ARRIVE guidelines and in accordance with the principles of the Basel Declaration. Previously, the animal research protocol was approved by the Ethics Committee of “Iuliu Hatieganu” University of Medicine and Pharmacy at Cluj-Napoca and the Veterinary Sanitary Committee of Cluj County, Romania (approval no. 85/19.07.2017).

In this study, Wistar albino male rats (N = 32) with a diaphyseal femoral fracture and enhanced bone consolidation, as was recently published by us [26], were analyzed with a predominant focus on the osseointegation evaluation using an intramedullary rod model of a rat femoral fracture. The animals were divided into four equal groups (N = 8/group) as follows: CG—control group of animals with standard (uncoated) Ti (STi) implants, which possessed a controlled surface roughness that was achieved using both grit-blasting and acid-etching methods; PESW group—STi implants and early HF-PESW stimulation for 2 weeks; BC group—BC-coated STi (BCSTi) implants; BC-PESW—BCSTi implants and HF-PESW stimulation.

At the beginning of the experiment, two-month-old rats that weighed 226 ± 13 g were anesthetized with an intramuscular cocktail of 2% xylazine and 10% ketamine. After the surgical field was prepared with an antiseptic solution, a transverse fracture of the femoral diaphysis was performed through a lateral approach of the thigh. Afterward, through a longitudinal incision at the knee level, uncoated Ti implants, STi implants that were 20 × 1 mm (i.e., CG and PESW group) and Ti implants that were coated with the biomimetic composite (BCSTi; i.e., BC group, as well as the BC and HF-PESW (BC-PESW group)) were retrogradely introduced into the medullary canal of the femur. Finally, the subcutaneous plane and the tegument were sutured. A team of two orthopedic surgeons performed all the surgeries. The animals were kept in cages without food restrictions at a temperature of 22 °C in a controlled environment with a day/night cycle of 12 h. Non-thermal HF-PESW stimulation (Diapulse^®^ Corporation of America, Great Neck, NY, USA) was used for the PESW and BC-PESW groups for 10 min/day daily for 2 weeks at a frequency of 400 pulses/s. The average power was 25.35 W, with a total energy of 15.21 kJ.

Anesthetic overdose was used to perform the animal euthanasia at 2 weeks (N = 16, N = 4/group) and at 8 weeks (N = 16, N = 4/group). Afterward, the left femur was carefully taken (e.g., using a lift-out technique) to avoid destroying the bone callus and it was placed in 10% formaldehyde for novel investigation.

### 2.4. Bone Markers: Alkaline Phosphatase and Osteocalcin

About 0.6 mL blood/examination was harvested from the retro-orbital sinus of each rat, at the beginning of the experiment (N = 32, initial time point), at two weeks (N = 32) and at 8 weeks (N = 16). Commercially available ELISA kits (OCN, Rat OC/BGP (Osteocalcin) ELISA kit, Cambridge, UK) and (ALP, ALP Reagent on Beckman Coulter AU, Brea, CA, USA) were used. The values were expressed as a percentage, starting from the initial values at time zero of the control group, expressed as 100% ± SD. The subsequent reports at two and eight weeks, respectively, were compared with these values. Thus, a clearer picture of the dynamics of these bone markers was obtained at the key moments of this study.

### 2.5. Advanced Micro-CT Approach

After the time of sacrifice, the left femoral bones with implants (N = 8/group) were scanned using a Bruker SkyScan 1172 micro-CT system (Kontich, Belgium) with a pixel size of 13.56 μm, X-ray energy level of 80 kV and a current of 100 μA. All data were exported to CTAn v1.17 (Bruker micro-CT, Kontich, Belgium) and CTVol v2.0 (Bruker micro-CT, Kontich, Belgium) for evaluation. The volume of interest (VOI) was established at 2 mm below the growth plate, with a height of 1 mm (75 slices) and a ring of 0.3 mm diameter around the implant. Multilevel thresholds from 225–700 was applied to discriminate the bone and calcified cartilage (225–330), dense cortical bone (331–700) and non-mineralized tissue (<225). The outcome variables were the bone volume percent (BV/TV), the mean trabecular number (Tb.N), the mean trabecular thickness (Tb.Th), the mean trabecular separation (Tb.Sp) and the BIC (bone-to-implant contact), which is the percentage of the area of the total implant surface that is covered by bone, according to Choi et al. [43].

### 2.6. Hematoxylin and Eosin (H&E) Staining Procedure: Histological Assessment of the Osseointegration

Bone callus evolution and osseointegration achievement at the fracture level were assessed using optical microscopy on the hematoxylin and eosin (H&E)-stained tissue samples from each of the four studied groups at two and eight weeks post operation. For the first twenty-four hours, the harvested femurs were fixed in a 4% paraformaldehyde solution. In order to obtain the decalcification of the bone samples, a 5% nitric acid solution was used, which was continuously stirred and changed daily for 10 days. The decalcification process was considered completed when the testing surgical blade easily penetrated the bone sample/or had the consistency of rubber. Samples were then dehydrated in increasing degrees of alcohol (50%, 75%, 100%), cleared in xylene (for alcohol removal) and embedded in paraffin. Afterward, cross sections with a 5 µm thickness mounted on a glass slide were deparaffinized, rehydrated and then stained with hematoxylin and eosin stain. For the histological examination, a Leica DMD 120 optical microscope was used, and the slides were examined and photographed.

At two weeks post operation, the stage of bone callus formation and its evolution were analyzed, describing the presence of any inflammatory response and soft callus formation. At eight weeks post operation, the formation and resorption of newly formed bone and the degree of osseointegration were evaluated in terms of the formation of cortical and trabecular bone at the fracture site [44,45]. Furthermore, the presence of osteoblasts, osteocytes and osteoclasts, as well as the bone remodeling and osteocytic network, were identified close to the implant surface.

### 2.7. Bone–Implant Interface

At the end of the decalcification process, the implants were removed with great care to prevent the destruction of the newly formed bone tissue around the implant. Afterward, samples of the intact bone–implant interface were used for scanning electron microscopy investigations of the retrieved implants at 8 weeks after the implantation surgery. The structural and chemical composition analysis of the bone–implant interface was achieved using FE-SEM images and EDX (energy-dispersive X-ray) spectra. Therefore, both the line profiles across the interface (cross-section profiles) and elemental composition mapping of the different interfacial zone were investigated. Moreover, the EDX spectra were used to examine the Ca/P molar ratio of bioapatite (e.g., the type of hydroxyapatite and the state of mineralization) in newly formed bone.

### 2.8. Statistical Analysis

Statistical analysis was performed using GraphPad Prism 6 for Windows. The values of the bone marker concentrations were converted to percentages and were defined as the mean value ± standard deviation (SD). The surface roughness parameters Ra and Rq (RMS) were determined using AFM [41] and are given as mean values ± standard deviation (SD) for the four implants: Ti, STi, PBC-STi and BCSTi implants. Statistical significance was identified using a one-way ANOVA test followed by Tukey’s post hoc test.

## 3. Results

### 3.1. Topography and Surface Structure of Modified Ti Implants

**Figure 1:**
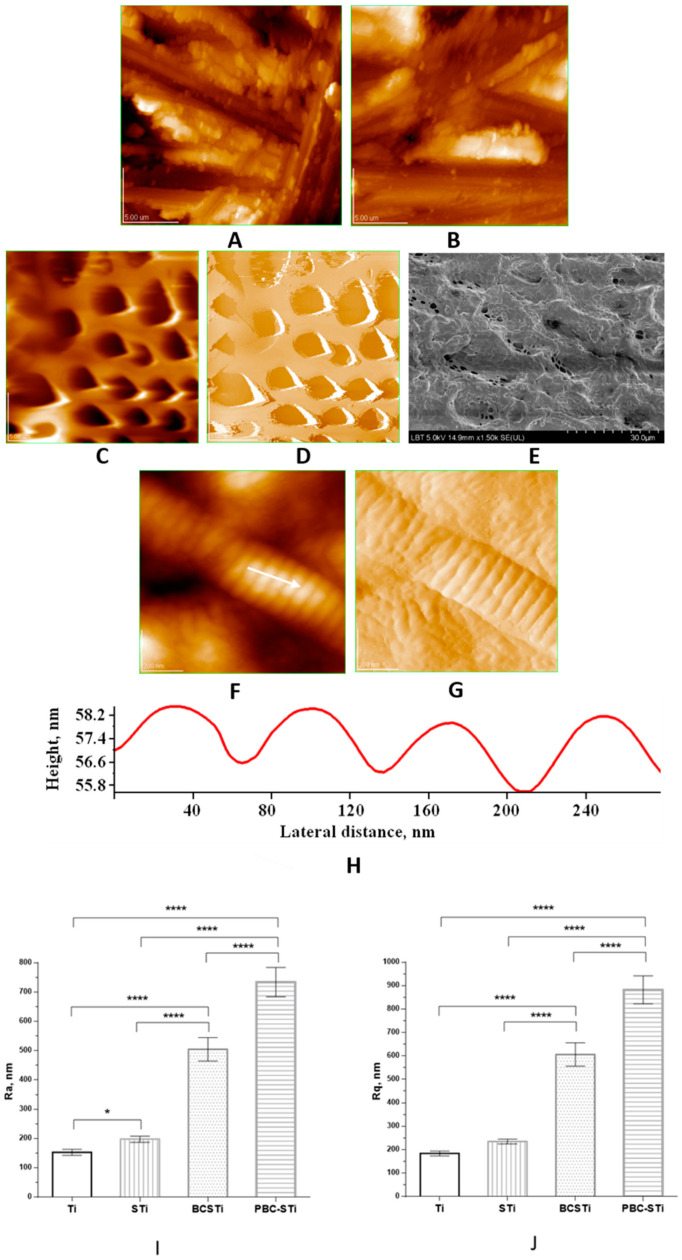
AFM images of the 2D topography of the modified surface of Ti implants via (**A**) grit blasting to create a Ti implant; (**B**) grit blasting and acid etching to create a standard (control; STi) implant. (**C**) The 2D topography and (**D**) amplitude image of a porous biomimetic composite (PBC) that was made of ms-HAP/COL@PLA and deposited on the surface of an STi implant, resulting in a PBC-STi implant; (**E**) SEM image of a biomimetic composite (BC) coating that was made of ms-HAP/COL@PLA/COL (having the outermost layer of self-assembled collagen (COL)), deposited on the surface of an STi implant, resulting in a BCSTi implant. (**F**) The 2D topography and (**G**) amplitude image of COL fibers that were adsorbed on a PBC (C,D) coating, resulting in a BCSTi implant (**E**–**G**); cross-section profile of a COL fiber (**H**) along the white arrow in panel (**F**), which illustrates the periodicity of the nanostructure of about 67 nm on the COL fiber. (**A**–**D**) scanned area of 20 µm × 20 µm; (**E**) scale bar of 30 µm; (**F**,**G**) scanned area of 1 µm × 1 µm. Ra (**I**) and Rq (**J**), respectively, of the four modified Ti implants: the surface of Ti implant that was modified using grit blasting, noted as Ti, followed by acid etching, named STi, and then coated with porous biomimetic composite, noted as PBC-STi, or coated with biomimetic composite, noted as BCSTi; * statistically significant with *p* < 0.05; **** statistically significant with *p* < 0.0001.

The topography and surface structure of the modified Ti implants were investigated using AFM images, as given in Figure 1, specifically for the Ti implant in Figure 1A; the control STi implant in Figure 1B; the coated STi implant with the porous biomimetic composite (PBC) ms-HAP/COL@PLA, resulting in the PBC-STi implants in Figure 1C,D; BCSTi implants, where the BC coating contained an outermost COL layer, specifically ms-HAP/COL@PLA/COL, were examined using FE-SEM and the associated image is given in Figure 1E; the morphology of the COL layer, particularly of the COL fiber, was visualized using AFM images and is given in Figure 1F,G; and the ultrastructure of the COL fiber is shown in the cross-section profile in Figure 1H. The surfaces of these implants were also characterized using the surface roughness parameters [41], namely, Ra and Rq (RMS), which were obtained using AFM. The Ra and Rq values and their standard deviations (SD) are given in Figure 1I,J for a scanned area of 20 μm × 20 μm.

The surface roughness parameters Ra and Rq were found to be the following: Figure 1A—Ra = 153 ± 10 nm and Rq = 184 ± 10 nm for the Ti implant, Figure 1B—Ra = 198 ± 10 nm and Rq = 235 ± 10 nm for the STi implant, Figure 1C—Ra = 734 ± 50 nm and Rq = 882 ± 60 nm for the PBC-STi implant and Figure 1E—Ra = 505 ± 40 nm and Rq = 606 ± 50 nm for the BCSTi implant. The thickness (height) of the biomimetic coating was about 1500 ± 60 nm; it was determined using AFM for the biomimetic composite deposited on the BCSTi implant (Figure 1E).

Figure 1 presents the ultrastructural arrangements of the various implant design features as follows: grit-blasted Ti implant (A); grit-blasted and acid-etched (control) STi implant (B); PBC-STi implant (C,D); and BCSTi implant (E,F,G,H); and surface modifications at the micron, submicron and nano levels, as well as the surface roughness (I,J). The results showed significantly higher Ra and Rq values in all groups compared to the Ti implants (about 150 nm), with considerably higher values in the case of the BCSTi (about 500 nm) and PBC-STi (around 750 nm) implants. Regarding the Rq values, for the BCSTi and PBC-STi implants, they were 3 and 4 times higher, respectively, compared with the Ti rods.

### 3.2. Alkaline Phosphatase and Osteocalcin vs. Animal Groups at Various Time Points

**Figure 2:**
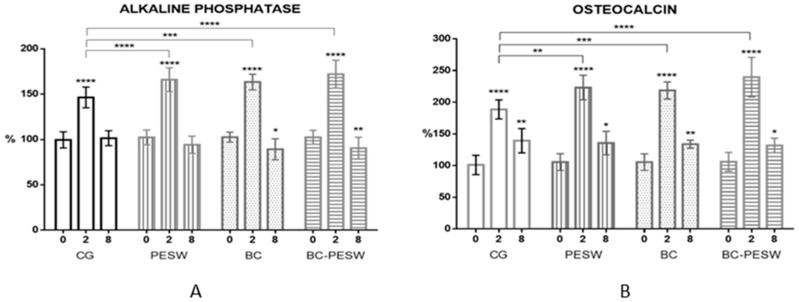
(**A**,**B**) Bone marker serum concentrations (alkaline phosphatase and osteocalcin) at zero, two and eight weeks post surgery. * statistically significant with *p* < 0.05; ** statistically significant with *p* < 0.01; *** statistically significant with *p* < 0.001; **** statistically significant with *p* < 0.0001.

According to Table 1, a significant increase in alkaline phosphatase (ALP) concentration (Figure 2A) was observed in all groups at 2 weeks compared to the initial values (*p* < 0.0001). The most significant increase was in the BC-PESW group (78%) when both consolidation methods were used, followed by the PESW group (69%) and the BC group (65%). In all these three groups, the values were higher compared to the control group, where the increase was 44% (*p* < 0.001).

At 8 weeks, the ALP values had decreased in all groups (*p* < 0.0001), where they were approximately equal or even lower compared to the initial values (0 weeks). In the control group, ALP was 2% higher at 8 weeks, while in the PESW (11%), BC (13%) and BC-PESW (17%) groups, the values were lower compared to the values at the beginning of the experiment. At 8 weeks, statistically significantly lower ALP values were found in the BC (*p* < 0.05) and BC-PESW (*p* < 0.01) groups compared with the initial values.

The osteocalcin (OCN) values (Figure 2B) increased significantly in all groups compared with the initial values, with the highest increase of 136% in the BC-PESW group, followed by 118% in the PESW group and 117% in the BC group, while in the control group (CG) growth was less than 89%. Furthermore, the values in the control group at two weeks were significantly lower compared to the other groups (*p* < 0.01 for the PESW and BC groups and *p* < 0.0001 for the BC-PESW group). At eight weeks, the OCN expression was significantly lower in all groups compared to the values at two weeks, however 32–40% higher than the initial values at 0 weeks, with statistically significant differences for all groups (*p* < 0.05).

### 3.3. Micro-CT Examination

The quantitative results of the osseointegration assessed using micro-CT are given in Table 2 for the implants used in the four groups of rats.

Significant differences were found between the BC and BC-PESW groups compared to the control group regarding the bone volume, trabecular thickness, trabecular separation and bone-to-implant contact (*p* < 0.05).

**Figure 3:**
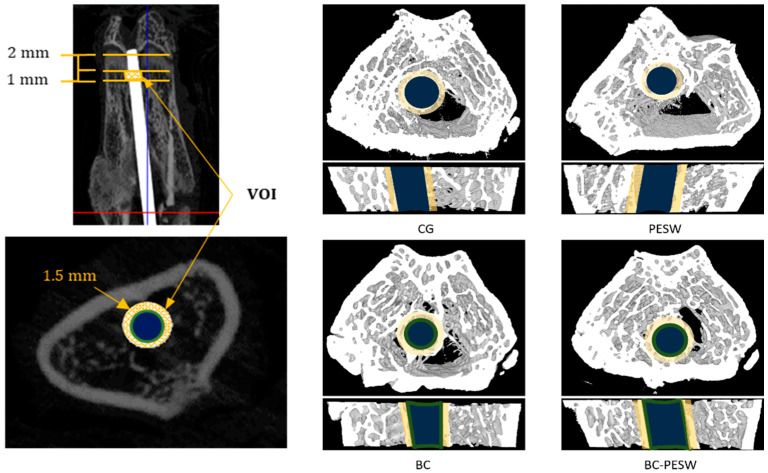
Three-dimensional reconstruction of the micro-CT images of the distal femur eight weeks after surgery. It gives examples of the cross-sections and 3D reconstructions of the implants and bone tissue around the implant. Dark blue—titanium implant; green—biomimetic composite (layer of 0.2 mm) around the titanium implant in the BC and BC-PESW groups; yellow—volume of interest (VOI) around the implant (0.3 mm); white and grey—bone.

New bone formation around the implant can be evaluated using micro-CT examinations (Figure 3), with the advantage of being non-invasive [43,46] and it gives similar results to those observed in histological examinations. The BIC evaluated using micro-CT analysis showed a significantly higher bone-to-implant contact in the group treated with titanium implants covered with biomimetic composites (with or without PESW stimulation) compared to the control group or the group with only PESW therapy.

### 3.4. Histological Assessment

**Figure 4:**
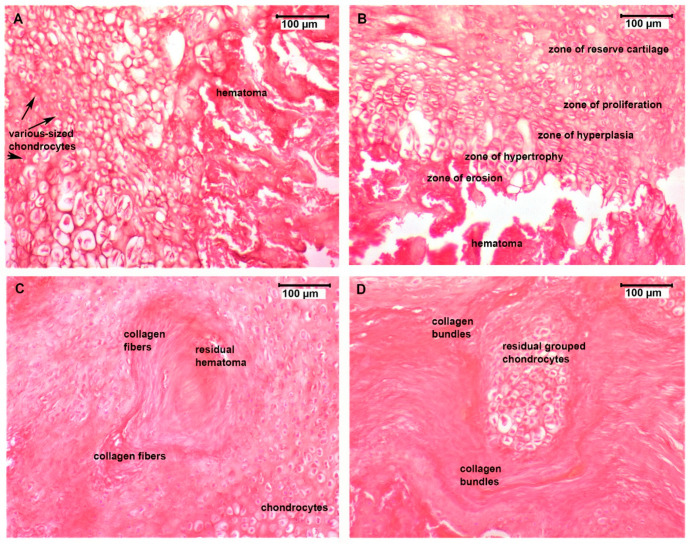
Optical microscopy images of H&E-stained slides of tissue samples at two weeks post surgery. (**A**) CG, 20×, various-sized chondrocytes indicating different stages of secretion activity and a high quantity of clotted blood and inflammatory cell recruitment. (**B**) PESW group, 20×, chondrocyte disposal is characteristic for endo-cartilaginous bone formation initiation with a zone of reserve cartilage, zone of proliferating chondrocytes, zones of hyperplasic and hypertrophic chondrocytes and a zone of erosion with an empty chondroplast hematoma was still present but to a lesser extent. (**C**) BC group, 20×, small chondrocytes in chondroplasts that were embedded in cartilaginous matrix with a different disposition of the newly synthesized collagen fibers. (**D**) BC-PESW group, 20×, residual grouped chondrocytes on a few microscopic fields that were contained in a large cartilaginous matrix with thick collagen bundles.

Analysis of the H&E-colored slide analysis of two-week post-operation tissue samples (Figure 4) revealed distinct histological differences between the four groups. In the control group (Figure 4A), tissue samples displayed marked signs of an initial acute inflammatory response due to a fracture hematoma as a result of the blood released from broken or torn vessels in the periosteum and medullary cavity after the fracture. The fibroblasts in the granulation tissue began to form cartilage progenitor cells that differentiated into chondrocytes with different sizes and an irregular disposition at the fracture site and were embedded in a provisional fibrin matrix. In the PESW group (Figure 4B), the inflammatory infiltrate was reduced, a hematoma was still present but to a lesser extent with clearance of necrotic tissue and the initiation of soft callus formation. Chondrocytes showed a particular zonal arrangement in the process of fibrocartilage production via endochondral ossification. Reserve cartilage chondrocytes were initially evenly dispersed (reserve cartilage zone); then, they started to proliferate and rearrange in axial isogenous groups (zone of hyperplasia). The chondrocytes became enlarged and were hypertrophic secondary to glycogen accumulation in their cytoplasm (zone of hypertrophy), they underwent apoptosis, and the immature extracellular matrix became calcified. In the erosion, the lacunae there was vascular ingrowth, with the recruitment of osteoprogenitor cells that initiated the process of creating an osteoid (immature bone)-on-cartilage scaffold. The BC group’s (Figure 4C) slide evaluation revealed a discrete residual post-traumatic hematoma and an advanced stage in the bone repair process, with small residual chondrocytes that were surrounded by a newly formed matrix rich in collagen-type fibers with an irregular disposition. The BC-PESW tissue samples (Figure 4D) revealed distant foci of residual grouped chondrocytes that were separated by thick collagen bundles embedded in a large eosinophilic matrix.

**Figure 5:**
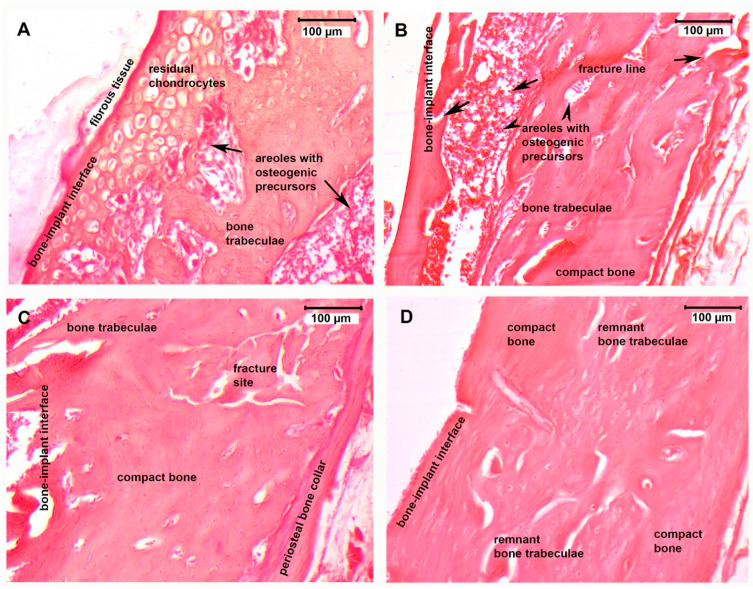
Optical microscopy images of H&E-stained slides of tissue samples at bone–implant interface near the fracture site at eight weeks post surgery. (**A**) In the control group, fibrotic tissue and residual cartilaginous tissues were identified in proximity with the titanium intramedullary implant, indicating a transition from cartilaginous precursors to incipient bone trabeculae formation. (**B**) The PESW group slides showed complete resorption of the fibro-cartilaginous tissue structures, which appeared irregularly disposed and less defined, but with many bone trabeculae at the fracture site. (**C**) BC group—fewer and well-defined bone trabeculae, with an osteoblast lining on their surface. Clear delimitation of the areole between the trabeculae with areas of compact lamellar bone deposition. On the lateral right side, the periosteal collar bone that formed during fracture healing can be seen. (**D**) The most advanced stage of bone remodeling with osseointegration of the titanium implant was seen in the BC-PESW group, where the trabecular bone tended to be replaced by compact bone with a lamellar disposition of bone matrix and osteocytes around Haversian canals. The bone–implant interface showed a compact bone cortex with active osteoblasts (they were round nuclei) near the medullary cavity.

Eight-week post-fracture tissue sample analysis of the bone remodeling process at the fracture site displayed major differences between the four investigated groups (Figure 5).

In the control group (Figure 5A), there was fibrotic tissue at the bone–implant interface, which was presented as an intense eosinophilic band, indicating a lower osseointegration capacity of the titanium rods in this group. Residual cartilaginous tissue indicated a transition from cartilaginous precursors to incipient bone trabeculae formation but at an initial phase of the bone remodeling process. The PESW group’s slides (Figure 5B) displayed complete resorption of the fibro-cartilaginous tissue structures, with the appearance of an irregular disposition and ill-defined bone trabeculae at the fracture site, surrounding areoles filled with new bone precursors. The BC group’s slides (Figure 5C) revealed a better remodeling of bone tissue during the titanium osseointegration process compared to those of the PESW group. The bone trabeculae were fewer but well defined, with osteoblasts lining their surface and clear delimitation of the areole between the trabeculae, with areas of compact lamellar bone deposition. The periosteum contribution to fracture healing through forming a periosteal collar bone around the fracture area was well expressed in the BC group’s slides. The BC-PESW group’s slide analysis (Figure 5D) showed no sign of implant rejection. The most advanced stage of bone remodeling with osseointegration of the titanium implant was seen in this group, where the trabecular bone tended to be replaced by compact bone with a lamellar disposition of the bone matrix and osteocytes around the Haversian canals. The bone–implant interface showed a compact bone cortex with active osteoblasts (they displayed round nuclei) near the medullary cavity.

### 3.5. Fine Structure of the Bone–Implant Interface

**Figure 6:**
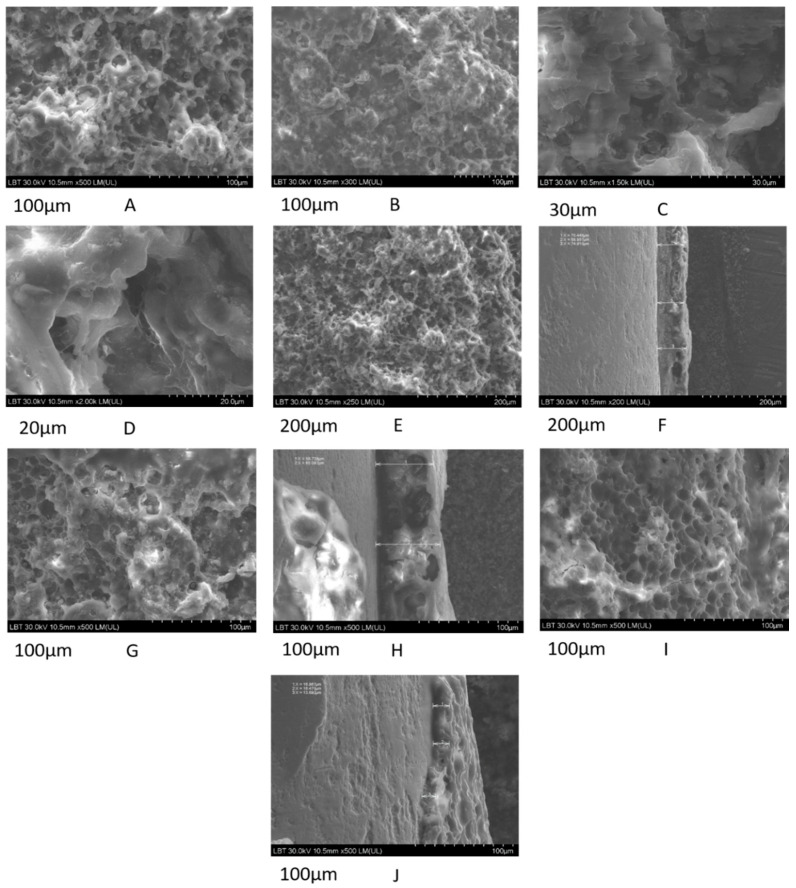
FE-SEM images for the bone–BCSTi implant interface without biophysical stimulation. Micro- and nanoscale structure of the retrieved implants (**A**–**E**) and the cross-section profile (**F**); FE-SEM image for the bone–BCSTi implant interface with HF-PESW biophysical stimulation (**G**) and the cross-section profile (**H**); FE-SEM image for the bone–STi implant interface with HF-PESW biophysical stimulation (**I**) and the cross-section profile (**J**). The scale bar sizes are 100 µm (**A**,**B**,**G**–**J**), 30 µm (**C**), 20 µm (**D**) and 200 µm (**E**,**F**).

Considering the histological features of the mineralized tissue and the osseointegration of the implants, particularly at 8 weeks after the implantation surgery, the FE-SEM images were used to further explain the bone growth and to analyze the structure and amount of bone that developed around the implants. Consequently, FE-SEM images of the retrieved STi and BCSTi implants were obtained for the four studied animal groups, namely, CG, PESW, BC and BC-PESW, at 8 weeks after implantation (Figure 6).

The representative FE-SEM images that were obtained of the retrieved modified Ti implants showed significantly higher amounts of newly developed bone on the BC-coated STi implants without (Figure 6A–F) and with biophysical stimulation using HF-PESW (Figure 6G,H) than for the control and STi (uncoated) implants with HF-PESW stimulation (Figure 6I,J). The osseointegration for STi without biophysical stimulation was rather poor, which was also found through the histological analysis. Consequently, the FE-SEM image is not shown for the control group of animals.

Further, it must be emphasized that the bone–implant interface is a complex zone that consists of mineralized, partially mineralized, and unmineralized areas [47]. The structural basis of the bone–implant interface is formed by mineralized collagen fibrils. In addition, non-collagenous macromolecules, such as osteopontin, osteocalcin and bone sialoprotein, can also be present in this complex zone.

The FE-SEM image analysis revealed greater amounts of bone growth on retrieved implants in the BC and BC-PESW animal groups with the biomimetic-composite-coated (BCSTi) implants than in the PEWS animal group with (noncoated) STi implants. This situation revealed strong osseointegration in the BC and BC-PESW animal groups.

Furthermore, significantly higher values were obtained for the thickness of the newly formed bone on the BCSTi implants, namely, 71 ± 3 µm without HF-PESW stimulation (Figure 6F) and 62 ± 3 µm with HF-PESW stimulation (Figure 6H), compared with the 15 ± 2 µm observed for (uncoated) STi implants with HF-PESW stimulation (Figure 6J), at 8 weeks after implantation.

For the Ti implant surfaces, the osseointegration was significantly greater for BC-coated STi implants than for the uncoated STi implants. The results of this in vivo study suggested that the presence of the biomimetic composite coating on the Ti implant’s surface enhanced the osseointegration, despite the similarities in the surface roughness values (Ra and Rq) of the implant surface at the nanoscale. This situation revealed strong osseointegration in the BC and BC-PESW animal groups.

The micro- and nano-architectures of the newly formed bone around the implants could also be observed in the FE-SEM images. As shown in Figure 6A–I, the pore sizes ranged from 10 to 120 μm (the biggest pores are not shown). This porous structure is suitable for cell attachment, cell migration and nutrient transport. In addition, collagen fibers that were mineralized with round nanoparticles were also observed.

**Figure 7:**
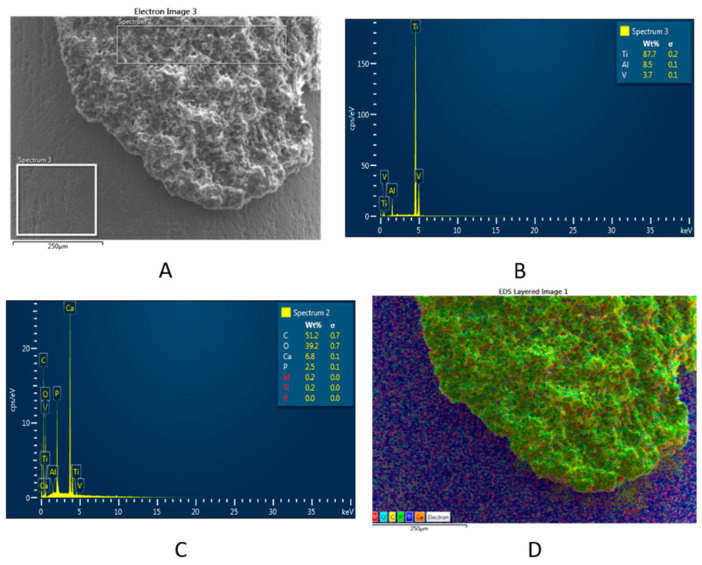

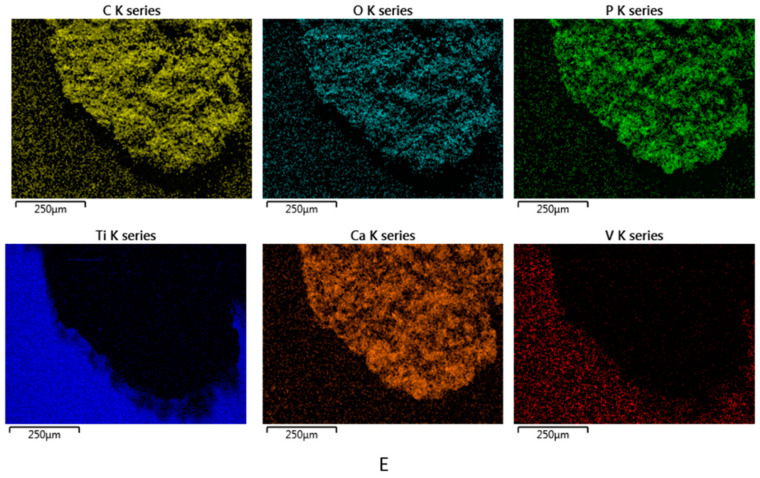
(**A**) FE-SEM image and EDX spectra of the (**B**) chemical composition of the Ti implant surface and (**C**) chemical composition of the bone–BCSTi implant interface with HF-PESW biophysical stimulation. (**D**) Multicolor distribution map showing the elements jointly on the FE-SEM image, followed by the distribution maps of individual elements (**E**), as given by the EDX spectra (**B**,**C**).

**Figure 8:**
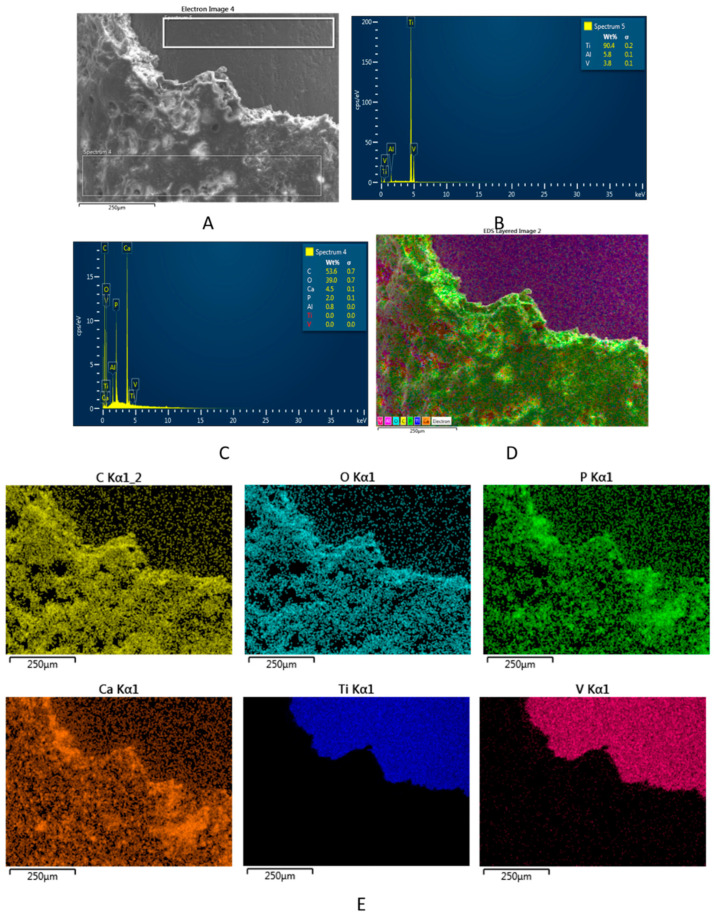
(**A**) FE-SEM image and EDX spectra of the (**B**) Ti implant surface and (**C**) bone–BCSTi implant interface without HF-PESW biophysical stimulation. (**D**) Multicolor distribution map showing the elements jointly on the FE-SEM image, followed by the distribution maps (**E**) of the individual elements.

The structure of the new mineralized bone tissue in direct contact with the modified Ti implants, such as STi and BCSTi, with and without biophysical stimulation in HF-PESW, was demonstrated using FE-SEM images (Figure 6) and using FE-SEM images and EDX spectra (Figure 7 and Figure 8).

The structure and chemical composition of the bone–BCSTi implant interface with the HF-PESW biophysical stimulation is given in Figure 7, which is representative of the BC-PESW animal group at 8 weeks after implantation in the intramedullary rod model of a rat femoral fracture. The SEM image in Figure 7A shows the structure of the newly formed bone on the BCSTi implant with HF-PESW biophysical stimulation. The EDX spectrum in Figure 7B gives the local chemical composition of the surface of Ti implants: about 87.7 wt% (Ti), 8.6 wt% (Al) and 3.7 wt% (V). The EDX spectrum in Figure 7C gives the chemical composition of the newly formed bone, which, expressed as Ca/P mole ratio, was about a 2.15 and illustrates a strongly mineralized surface with Ca in excess compared to stoichiometric HAP, which has a Ca/P mole ratio of about 1.67. The multicolor distribution map in Figure 7D shows all the elements (except Al) jointly on the FE-SEM image, indicating a homogeneous distribution of each element (Figure 7E), e.g., C and O, as primary elements of collagen fibers, as well as Ca and P as elements of bio-apatite in the newly formed bone.

In the following example, the structure and chemical composition of the bone–BCSTi implant interface is given in Figure 8, which is representative of the BC animal group. The SEM image in Figure 8A shows the structure of the newly formed bone on the retrieved BCSTi implant. The EDX spectrum in Figure 8B gives the local chemical composition of the surface of Ti implants, which was about 90.4 wt% (Ti), 5.8 wt% (Al) and 3.8 wt% (V). The EDX spectrum in Figure 8C gives the elemental composition of the newly formed bone, which revealed a Ca/P mole ratio of about 1.73, rather close to stoichiometric HAP, which is characterized by a Ca/P mole ratio of 1.67. The multicolor distribution map in Figure 8D shows all elements (except aluminum) jointly on the FE-SEM image, indicating a homogeneous distribution of each element (Figure 8E), e.g., C and O, as the predominant elements of collagen fibers as well as Ca and P, as elements of bioapatite in the newly formed bone.

For the BC and BC-PESW animal groups, the newly formed bone on the surface of the BCSTi implants was mainly mineralized by crystallites of stoichiometric hydroxyapatite with Ca/P mole ratios of 1.65–1.73 (1.69 ± 0.04) for the BC group and 1.83–2.15 (1.99 ± 0.16) for the BC-PESW group, as judged using FE-SEM images and EDX spectra. For the PESW group, in a similar way, collagen fibrils were mineralized by hydroxyapatite with Ca/P mole ratios of 1.30–1.60 (1.45 ± 0.15), while for the CG, the mineralization was rather poor. These results suggest that the in vivo performances of these orthopedic implants were in the following order: STi (CG) < STi (PESW) < BCSTi (BC-PESW) ≤ BCSTi (BC) for the modified Ti implants in the four animal groups. These findings provide evidence that the novel biomimetic composite of HAP-1.5 wt% Mg-0.2 wt% Zn-0.2 wt% Si/COL@PLA/COL was successfully designed.

This innovative BC coating enriched in Mg was developed to enhance the osseointegration of Ti implants and can be used for the realization of Ti orthopedic implants that possess a controlled structure and surface roughness, as well as a suitable chemical composition. The surface functionalization of orthopedic Ti implants can be done by using an appropriate layer-by-layer (dip-coating) method.

## 4. Discussion

An in vivo evaluation of the osseointegration of two different surfaces of STi and BCSTi implants was required for a profound understanding of the role of surface features and chemical composition of coatings on a Ti implant surface. In the present investigation, an early (in the first two weeks from surgery) HF-PESW biophysical stimulation was also used. Accordingly, the fine structure and chemical analysis of the bone–implant interface, both in the absence and the presence of HF-PESW, was also important for a deeper understanding of the relationships between the structure and biological function of nanostructured modified Ti implant surfaces.

Figure 1 shows the surface structure and topography of four different implant surfaces: Ti (A: grit-blasted), STi (B: grit-blasted and acid-etched, standard implant), PBC-STi (C,D: STi coated with a porous biomimetic composite (PBC)) and BCSTi (E,F,G,H: STi coated with BC containing an adsorbed COL outermost layer), which were all prepared using a layer-by-layer method.

The morphology of the biomimetic composite ms-HAP/COL@PLA was investigated using AFM (Figure 1C,D), which showed that the ms-HAP/COL NPs were well dispersed in the PLA matrix. The AFM images (Figure 1C,D) also illustrated that the surface of the biomimetic composite was highly porous, and no aggregated NPs of ms-HAP/COL in the PLA matrix were revealed. The porous biomimetic composite ms-HAP/COL@PLA, noted as PBC, was deposited on the surface of an STi implant, resulting in a PBC-STi implant with a controlled surface topography.

Finally, the PBC-STi implant was subsequently covered by an adsorbed self-assembled COL layer, obtaining the biomimetic composite (BC) ms-HAP/COL@ PLA/COL, which was deposited on STi implants, named BCSTi implants. Thus, the layer-by-layer self-assembly method was found to be beneficial to the formation of a biomimetic composite layered on Ti implants. The thickness of about 1500 ± 50 nm was determined using AFM for the biomimetic coating (BC) containing the adsorbed COL layer on the surface of the BCSTi implants (Figure 1E–G).

The surface roughness parameters Ra and Rq (RMS) were also determined using AFM for a scanned area of 20 μm × 20 μm and are given in Figure 1I,J. From these four implants with rather similar surface roughnesses, which were at the level of hundreds of nanometers, only two were chosen for implantation in the rat model, specifically the STi and BCSTi implants, after considering their potential biological benefits. For instance, the collagen self-assembled layer on the porous biomimetic composite (PBC) coating of the STi implant surface could influence the adsorption of proteins at the implant surface; the cell adhesion, proliferation and differentiation; and the development of newly formed bone at the implant surface. The STi implant surface that was covered by biomimetic coating became the BCSTi surface, which showed significantly higher roughness than the STi implant, but was still at the nanoscale level.

Further, in this study, four stable retrieved implants, namely, STi implants from the CG, STi implants with HF-PESW from the PESW group, BCSTi implants from the BC group, and BCSTi implants with HF-PESW from the BC-PESW group, were evaluated using histological, micro-CT, bone–implant contact (BIC) and bone–implant interface analyses at 8 weeks after implantation surgery.

The structural analysis of the bone–implant interface was performed by using FE-SEM images (Figure 6), as well FE-SEM images and EDX spectra (Figure 7 and Figure 8) on retrieved implants at 8 weeks after the implantation surgery.

Our study demonstrated significant osseointegration, which was characterized by a greater bone-to-implant contact for the biomimetic composite coated (BCSTi) implants with and without HF-PESW stimulation, as compared to the STi implants, which only had its surface grit-blasted and acid-etched. Certainly, the osseointegration was related to the design of the implant and implant surface properties, as well as the bone quality and quantity at the implantation site. The osseointegration of the implants was correlated primarily to their surface properties, which were determining factors for rapid and intimate bone–implant contact. Regarding the implant surface characteristics, the topography, surface roughness and chemical composition of its coating were found to be critical factors for osseointegration. These parameters could influence the development of newly formed bone at the implant surface. The surface quality of coated implants could be subdivided into surface nanostructures and chemical properties.

The topography and surface nanostructure of the four Ti implant surfaces, i.e., Ti, STi, PBC-STi and BCSTi implants, are given in Figure 1, which were produced using AFM and SEM images. The topographic properties of these Ti implant surfaces were evaluated in terms of the surface roughness at the nanoscale level between about 150 nm and about 900 nm. The chemical composition of the PBC-STi and BCSTi coatings on the STi surface was a decisive factor for the selection of BCSTi implants for the in vivo study, after also taking into account the potential biological effect of the ms-HAP/COL@PLA/COL biomimetic composite coating.

Further, the in vivo study showed a stronger bone response for the BCSTi implants than for the uncoated Ti implants while having a similar nanoscale roughness. This result exemplified the predominant role of the chemical composition of the coating layers on the STi surface of implants for the osseointegration process. The surface functionalization of the orthopedic Ti implants was achieved by using an appropriate layer-by-layer (dip-coating) method.

Furthermore, the thickness of newly formed bone on the retrieved BCSTi implants, determined using FE-SEM, was much higher (between 62 and 71 µm) than the thickness of about 15 µm that was found for the STi implants that were used in the HF-PESW stimulation. The increase in the thickness was statistically significant and suggests the BCSTi implants, with nanoscale rough surfaces, as the best option for potential clinical applications with or without HF-PESW stimulation.

These results showed that the in vivo effectiveness of these modified Ti implants were in the following order: STi (CG) < STi (PESW) < BCSTi (BC-PESW) ≤ BCSTi (BC), as assessed by the implantation in the rat femur in the four animal groups.

These findings provide evidence that the novel HAP-1.5 wt% Mg-0.2 wt% Zn-0.2 wt% Si/COL@PLA/COL biomimetic composite was successfully designed. This innovative BC coating, enriched in Mg, was developed to enhance the osseointegration of Ti implants and can be used for the realization of Ti orthopedic implants since they possess a controlled structure and surface roughness, as well as suitable chemical composition, which is particularly advantageous for osteoporotic bone and a low bone density.

The activity of alkaline phosphatase and osteocalcin are useful biochemical markers for assessing bone formation and they play an important role in bone mineralization. Osteoblasts, the bone-forming cells, express proteins on their surface, such as alkaline phosphatase isoforms, which can be derived from bone or non-specific tissue. Osteocalcin is the most abundant non-collagenous bone protein [48].

Through several molecular mechanisms, HF-PESW can stimulate bone cell activity by accelerating proliferation and cell differentiation [49]. HF-PESW increases the activity of osteoblasts and upregulates genes that are related to bone formation and matrix components. Moreover, it reduces the resorption activity of osteoclasts and downregulates the genes that are related to the degradation of the bone matrix components [50]. Studies on osteoblast cultures showed a marked increase in alkaline phosphatase after HF-PESW exposure of 20% for 7 days and 58% for 10 days of stimulation [51]. Furthermore, HF-PESW stimulates early osteogenic induction in vitro by modulating the ALP and OCN activity and bone matrix mineralization, independent of BMP-2 presence in mesenchymal stem cells (MSC) that are derived from bone marrow or adipose tissue. Regarding the dynamics of ALP growth, the group exposed to HF-PESW showed faster growth than the other groups until two weeks, after which, the ALP activity decreased [52]. Our results showed a significant increase in alkaline phosphatase during the HF-PESW stimulation in the bone consolidation process with a significant 69% increase over the initial values. Considering that the HF-PESW due to the generated electromagnetic field simulates the micromovements at the fracture focal point and favors callus formation, this explains the stimulation of alkaline phosphatase activity. In the case of the rats treated with BC coated titanium nails, the ALP levels were significantly increased after two weeks (65%) versus the control group (44%) and had a comparative efficiency with HF-PESW stimulation (69%). Hydroxyapatite, especially multi-substituted hydroxyapatite, creates a microclimate that is favorable for cellular recruitment, and due to its osteoconductive capacity, it stimulates the activity of osteoblasts and facilitates the bone strengthening that is demonstrated by higher ALP activity. Subsequently, between the 2nd and 8th weeks, the ALP activity reverted to approximately the initial values. The ALP values at eight weeks were significantly lower than the initial values (0 weeks) in groups where titanium intramedullary nails coated with multi-substituted hydroxyapatite were used, with or without HF-PESW stimulation. The marked decrease in ALP activity indicated an increase in fracture focal stability and a more advanced stage of bone consolidation, which was more evident in these groups.

Osteocalcin is a late marker of bone formation and it is more specific than ALP, regarding the changes of bone metabolism [53]. Alkaline phosphatase maintained its increased levels for a shorter period of time, being less specific than OCN, but nevertheless it is an efficient marker of bone turnover in case of fracture healing. ALP and OCN activity increased significantly at two weeks in all groups, compared to initial values, with the highest increase in the BC-PESW group, followed by the BC and PESW groups. In the CG, the increase of OCN values was less than 89% in the first two weeks, significantly lower compared to the other groups. At eight weeks there is a significant decrease in OCN expression, however 32–40% higher compared to the initial values (*p* < 0.05). HF-PESW stimulation, as well as titanium nails coated with multi-substituted hydroxyapatite–collagen, determined significantly higher OCN expression values than the control group at two weeks (*p* < 0.01). Furthermore, concomitant use of the two methods had the strongest effect on the OCN expression (*p* < 0.0001 vs. CG). In normal conditions, OCN is secreted by osteoblasts, causing changes in the expression of this protein in the final stages of osteogenic differentiation. In vitro studies showed that OCN expression on days 21–28 was even higher than the ALP activity, after HF-PESW stimulation, showing that the pulsed electromagnetic field had an impact in the initial stages of osteogenic differentiation, which is maintained throughout all this period [52]. Osteocalcin causes an earlier onset of the bone remodeling process by activating both osteoblasts and osteoclasts during early bone formation process, demonstrated in studies where cylindrical nanocrystalline hydroxyapatite/collagen implants were introduced in the tibia of Wistar albino rats, enhancing bone formation and regeneration [54]. 

Consequently, the dynamics of ALP activity and OCN expression showed, in the groups that were exposed to HF-PESW and treated with multi-substituted hydroxyapatite–collagen-coated titanium intramedullary nails, higher values, with an intense osteoblastic activity. Considering that ALP is an early bone formation marker, its activity diminished at eight weeks, when the fracture was stabilized by the newly formed trabecular bone. On the other hand, as a late bone formation marker, the OCN values remained higher, even after eight weeks.

Three-dimensional micro-CT imaging determines a global image of the bone-implant interface, which can supplement the drawbacks of the BIC histomorphometric measure, representing a useful complementary tool in the quantitative measurement of bone–implant interface analysis [43]. This method is non-invasive, does not destroy the tissue and the samples, and gives a complete three-dimensional image with a resolution at the micrometer level. It allows for measuring the parameters for quantitative and qualitative evaluation of the tissue, peri-implant bone formation and bone–implant interface. In our study, we included 75 slices with a height of 1 mm and 0.3 mm around the implant, in which we evaluated the percentage of bone tissue, the number and average thickness of the bone trabeculae and the distance between them [55]. Moreover, using the formula recommended by Choi et al. [43], we calculated the bone-to-implant contact percent. Micro-CT that is used for the evaluation of osseointegration has comparable results to the histological method, providing a very useful and non-invasive tool in the calculation of these parameters [56].

The quantitative results of the implant osseointegration using advanced micro-CT assessment showed more peri-implant bone tissue in all groups compared to the control group. The results are summarized in Table 2 and show that the groups that were treated intramedullary with Ti rods coated with a biomimetic composite (BC) with or without HF-PEWS stimulation were the most efficient and had the strongest effects on all micro-CT parameters. Micro-CT quantitative results revealed that the multi-substituted hydroxyapatite and collagen coating produced an anabolic effect on the bone around the implant, with a significant increase in peri-implant bone volume percentage, trabecular number and trabecular thickness, and significantly decreased the trabecular spacing, compared to the control group. The Ti rods that were coated with the biomimetic composite increased the trabecular homogeneity of the peri-implant bone, which is essential for the long-term success of the implant [57]. The effect of the HF-PESW was minimal on bone formation around the implant with significantly increasing trabecular thickness compared to the CG.

Our study showed that the combination of HF-PESW and titanium intramedullary nails that were coated with ms-HAP/COL@PLA/COL containing multi-substituted hydroxyapatite enriched in Mg, namely, HAP-1.5 wt% Mg-0.2 wt% Zn-0.2 wt% Si, and collagen exhibited the best properties for implant osseointegration, although there were no substantial differences compared to intramedullary nails that were coated with BC used alone. Similarly, in the case of ovariectomized rats, good results were demonstrated regarding the osteointegration of related materials, such as strontium-doped brushite coating [58] and strontium-substituted hydroxyapatite coating [59] on implants. The bone-to-implant contact was 48% when using the biomimetic composite coating, and in its concomitant use with HF-PESW, the BIC values were up to 54% compared to the control group (21%). These results suggested that the use of the biomimetic composite coating, in combination with HF-PESW, had beneficial effects on anchoring the implant to the surrounding bone tissue, facilitating the process of osseointegration. Correlated with other micro-CT parameters, histological results and FE-SEM images, the composite biomimetic material improved the anchoring of the implant to the surrounding bone tissue, which led to increased osseointegration and implant stability.

Therefore, in this study, we evaluated 3D images using micro-CT to investigate the changes in implant surface and bone tissue reaction and supplemented this with 2D histological studies to obtain cytological information about the bone formation around implants. Histological sections at eight weeks confirmed the direct bone–implant contact for all specimens. There was no evidence of fibrous tissue layers surrounding the biodegradable coating on the Ti implants at any time point, except the control group, where we found a thin layer of fibrotic tissue in proximity to the titanium intramedullary implant. In the BC and BC-PESW groups, the bone–implant interface showed a compact bone cortex with active osteoblasts near the medullary cavity with bone remodeling areas and marrow spaces (Figure 5C,D). Furthermore, in these two groups, the periosteal activity of osteosynthesis was better observed. The periosteum was well vascularized and had mesenchymal stem cells, which represented a good source of osteoprogenitor cells. The periosteum has a high regenerative capacity and significantly contributed to fracture healing by modulating the inflammatory process and promoting early bone union through forming a periosteal collar bone around the fracture (Figure 5C) [60]. Blood vessels of different sizes and an osteoid matrix were present inside the marrow spaces. Callus formation was influenced by the presence and stability of the fracture site. In these cases, the implant was surrounded by highly vascularized granulation tissue, and bone cell precursors appeared around the implant (Figure 5A,B). The appearance of the callus that predominantly consisted of hyaline cartilage that was rich in chondrocytes with osseous trabeculae (Figure 4C,D) suggested the enchondral ossification process in which the cartilage was progressively replaced by the woven bone. The fracture healing process followed a certain pattern, which could be influenced by the stability provided in the fracture site. The size of the callus was directly proportional to the degree of mobility in the fracture site, and with its maturation, a more organized structure of osteoblasts appeared that was aligned along the bone trabeculae [61]. These changes could also be seen on histological images at 8 weeks post implantation at the bone–implant interface, where osteoblasts were aligned around the medullary canal and compact bone was present (Figure 5).

Different studies showed the beneficial effects of titanium implants [62,63] and biomimetic composites based on hydroxyapatite [64,65], hydroxyapatite substituted with strontium [66] or dicalcium phosphate [67] in facilitating peri-implant bone formation and anchoring the implant to the surrounding bone tissue, which increases the stability and osseointegration. Micro-CT results, together with histological analysis, bone markers, AFM and FE-SEM evaluation, produced valuable and relevant information regarding the osseointegration of the metallic implants [68].

In our study, we used an innovative, biomimetic composite that was based on multi-substituted hydroxyapatite, HAP-1.5 wt% Mg-0.2 wt% Zn-0.2 wt% Si, collagen and PLA, which showed excellent effects regarding improving the osseointegration of implants without side effects, making it a material of interest with potential clinical use for long-term implant–bone fixation.

The BC coating, comprising Mg-Zn-Si-HAP and COL in a PLA matrix, on Ti implants could induce the early formation of a bone-to-implant interface [69,70], including the bone sites with osteoporosis, producing a surface treatment with high osteogenic potential due to the release of Mg, Zn and Si in vivo. Clearly, the BC coating on titanium implants promoted new bone formation in the Wistar albino rat model. Consequently, introducing Mg, Zn and Si in the HAP lattice simultaneously with COL and PLA in a biomimetic coating on Ti implant was an effective design strategy to increase the degradation rate of pure HAP [25], while simultaneously improving the bone integration ability of the modified implants. The synergic effect of releasing Zn, Si and, most importantly, Mg contributed to the best implant–bone integration.

## 5. Conclusions

This study was the first to report that layer-by-layer biomimetic composite coatings on Ti surfaces enhanced osseointegration and new bone formation. The major finding in this in vivo study suggested that the biomimetic composite ms-HAP/COL@PLA/COL, made of HAP-1.5 wt% Mg-0.2 wt% Zn-0.2 wt% Si, collagen and polylactic acid, can be used for orthopedic applications. The novel biomimetic composite was designed with a controlled structure and surface roughness and showed a strong performance in vivo by enhancing the osseointegration of Ti orthopedic implants and also sustaining the bone regeneration through an ion release process from the coatings. Moreover, the in vivo findings revealed a combined beneficial effect of micro- and nanoscale modifications of titanium surfaces, coupled with their biomimetic functionalization.

Therefore, our BC coating could be a reliable surface modification of orthopedic implants to enable the delivery of osteoinductive ions (such as Mg^2+^, Zn^2+^ and SiO_4_^4−^) with synergistic benefits of a biomimetic composite based on a polylactic acid matrix and collagen fibers. Thus, jointly introducing Mg, Zn and Si in the HAP lattice with COL and PLA in a biomimetic coating on a Ti implant is an effective design strategy to improve the bone integration ability of the modified metallic implants.

## Figures and Tables

**Table 1:** Bone marker serum concentration assessment: initial (0 weeks) and two and eight weeks post operation; * statistically significant with *p* < 0.05; ** statistically significant with *p* < 0.01; **** statistically significant with *p* < 0.0001 compared with the initial values (0 weeks) of the group; comparisons were performed within the same group relative to the initial value (0 weeks); alkaline phosphatase (ALP), osteocalcin (OCN).

Bone Marker	Time after Surgery	Animal Group			
		CG	PESW	BC	BC-PESW
ALP (%)	0 weeks	100 ± 9	103 ± 8	103 ±6	103 ± 7
	2 weeks	144 ± 11 ****	172 ± 7 ****	168 ± 8 ****	181 ± 11 ****
	8 weeks	102 ± 8	92 ± 8	90 ± 12 *	86 ± 9 **
OCN (%)	0 weeks	100 ± 15	105 ± 13	105 ± 13	106 ± 15
	2 weeks	189 ± 15 ****	223 ± 20 ****	222 ± 12 ****	242 ± 20 ****
	8 weeks	140 ± 19 **	136 ± 19 *	134 ± 12 **	132 ± 12 *

**Table 2:** Quantitative results of the implant osseointegration, as assessed using micro-CT; * *p* < 0.05 vs. CG; ^&^ *p* < 0.05 vs PESW. The bone volume per total volume (BV/TV), the mean trabecular number (Tb.N), the mean trabecular thickness (Tb.Th), the mean trabecular separation (Tb.Sp) and bone-to-implant contact (BIC) are given for the four animal groups.

Osseointegration Marker	CG	PESW	BC	BC-PESW
BV/TV (%)	24.4 ± 3.8	28.9 ± 4	36.8 ± 4.6 *^,&^	42.7 ± 6 *^,&^
Tb.N (1/mm)	154 ± 18	166 ± 20	180 ± 18 *	189 ± 20 *
Tb.Th (µm)	153 ± 13	174 ± 13 *	176 ± 17 *	195 ± 14 *^,&^
Tb.Sp (µm)	386 ± 44	353 ± 47	319 ± 39 *	287 ± 58 *^,&^
BIC (%)	21 ± 5	27 ± 7	48 ± 8 *^,&^	54 ± 11 *^,&^

## Data Availability

See Supplementary Material.

## Supplementary Materials

The following are available online at https://www.mdpi.com/article/10.3390/mi12111352/s1.
